# Familial adult myoclonic epilepsy (FAME): clinical features, molecular characteristics, pathophysiological aspects and diagnostic work-up

**DOI:** 10.1515/medgen-2021-2100

**Published:** 2022-01-12

**Authors:** Lorenz Peters, Christel Depienne, Stephan Klebe

**Affiliations:** Department of Neurology, University Hospital Essen, Essen, Germany; Institute of Human Genetics, University Hospital Essen, University of Duisburg-Essen, Essen, Germany

**Keywords:** familial adult myoclonic epilepsy, seizures, myoclonus, cortical tremor, repeat expansion, repeat insertion, intronic, AT-rich, long-read, molecular combing, fiber FISH

## Abstract

Familial adult myoclonic epilepsy (FAME) is a rare autosomal dominant disorder characterized by myoclonus and seizures. The genetic variant underlying FAME is an intronic repeat expansion composed of two different pentamers: an expanded TTTTA, which is the motif originally present at the locus, and an insertion of TTTCA repeats, which is usually located at the 3′ end and likely corresponds to the pathogenic part of the expansion. This repeat expansion has been identified so far in six genes located on different chromosomes, which remarkably encode proteins with distinct cellular localizations and functions. Although the exact pathophysiological mechanisms remain to be clarified, it is likely that FAME repeat expansions lead to disease independently of the gene where they occur. We herein review the clinical and molecular characteristics of this singular genetic disorder, which interestingly shares clinical features with other more common neurological disorders whose etiology remains mainly unsolved.

## Introduction

Familial adult myoclonic epilepsy (FAME) is an autosomal dominant disorder characterized by a cortical myoclonic tremor and generalized seizures. It was first described in 1990 in Japan [1] but has meanwhile shown a worldwide distribution [2]. Over the past decades many alternative names like benign adult familial myoclonic epilepsy (BAFME), familial cortical myoclonic tremor with epilepsy (FCMTE), familial cortical myoclonic tremor (FCMT), familial essential myoclonus and epilepsy (FEME), familial benign myoclonus epilepsy of adult onset (FMEA) and heredofamilial tremor and epilepsy (HTE) have been proposed, reflecting its clinical heterogeneity. The latter underlines the difficulty to clearly distinguish this clinical entity from more frequently occurring, clinically overlapping disorders [3], [4]. The exact prevalence of FAME remains largely unknown as this condition is probably often underdiagnosed, but it is estimated to be a rare disorder affecting less than one out of 35,000 individuals [5].

In the 20 years following its clinical description, several genetic loci have been mapped on different chromosomes, suggesting that FAME is genetically heterogeneous. However, recent evidence suggests that the cause of FAME is actually linked to the same pathogenic repeat expansion in different genes and that the pathophysiological mechanisms are likely independent from the gene where the expansion occurs or its functions. In this setting, FAME is becoming a paradigm illustrating how pathogenic variants may lead to the same disease irrespectively of their location in the genome.

In the present review, we give an overview of the genetics, clinical features, genotype–phenotype correlations and diagnostic work-up of FAME.

## Clinical presentation: predominant cortical myoclonus and epilepsy

FAME symptoms usually occur in the second or third decade with a highly variable age of onset (range, 3–70 years) [2]. Epidemiologic data about the penetrance are not available but approximately 150 families with FAME are reported so far [6]. It is assumed that there are many more FAME families that remain undiagnosed due to mild and/or overlooked symptoms or misdiagnosis with more frequent disorders. The disease course of FAME is usually slowly progressive with a disease duration over several decades [7], [8].

## Cortical myoclonus

The defining symptom and most prominent movement disorder of FAME is cortical myoclonus. Myoclonus itself is defined as a “complex hyperkinetic movement disorder characterized by sudden, brief, involuntary jerks of a single muscle or a group of muscles” [9]. The origin of myoclonic jerks can be peripheral, spinal, subcortical or cortical as in FAME patients [9]. The cortical myoclonus predominantly affects the distal upper limbs in posture or action, with a variable severity between and within pedigrees. However, it may also affect other body parts like lower extremities, head, trunk, proximal upper limbs and facial muscles (e. g., eyelids). Myoclonus is often provoked by a startle reaction and worsened by goal-directed movements. Markedly, the movements are occasionally interrupted by bigger myoclonic jerks predominantly in the upper limbs.

Clinically, due to its shivering-like small jerks and high frequency (approx. 10 per second) the irregularity is not easy to recognize; therefore, it is sometimes misdiagnosed as essential tremor (ET) (Table 1, Fig. 2) or enhanced physiological tremor. ET represents the most frequent movement disorder in neurology with a presumed prevalence of 0.4 % to 6.3 % worldwide [10] and frequently shows a positive family history [11]. In contrast to FAME, it has not yet been possible to find one or several genes linked to ET despite positive results in linkage analyses [12]. For clinicians it is therefore important to distinguish between FAME, other types of myoclonic jerks and ET. To ensure differentiation between ET and FAME, the term “tremor” should be replaced by rhythmic cortical myoclonus in FAME patients.

**Table 1:** Comparison between symptoms, EMG and therapy in essential tremor and cortical myoclonus.

Syndrome	Localization	Frequency	EMG pattern	Therapy	Other features
Essential tremor	UE with or without other locations (voice; LE), predominantly bilateral	4–12 Hz	rhythmic; commonly synchronized	Propranolol, Primidone, Topiramate	No further neurological signs (if further neurological signs occur [e. g., ataxia] the classification is “essential tremor plus”)
Cortical myoclonus	UE (unilateral or bilateral)	9–18 Hz	arrhythmic bursts of around 15 to 60 ms	AED (e. g., Levetiracetam, sodium valproate)	Cortical excitability (giant SEP, jerk locked EEG changes)

UE, upper extremities; LE, lower extremities; AED, antiepileptic drugs; Hz, hertz; SSEP, somatosensory-evoked potentials; EEG, electroencephalography

**Table 2:** List of genes containing pathogenic TTTTA/TTTCA repeat expansions related to FAME.

Chr.	Gene name	Intron	Genomic coordinates (hg38)	Normal alleles_repeat range_	Pathogenic alleles_repeat range_	Ref.
2	*STARD7*	1	96197067–96197121	TTTA(TTTTA)_9–20_TTTT	(TTTTA/TTTCA)_661–735_	[14]
3	*YEATS2*	1	183712192–183712226	TTTTATGTTC(TTTTA)_7_TTTTTT	(TTTTA/TTTCA)_exp_	[27]
4	*RAPGEF2*	14/20*	159342529–159342618	(TTTTA)_5_(TATTA)(TTTTA)_12–14_	(TTTTA/TTTCA)_exp_	[23]
5	*MARCHF6*	1	10356339–10356411	TTTTTTATTTA(TTTTA)_10–30_TTTT	(TTTTA/TTTCA)_660–2,800_	[7]
8	*SAMD12*	4/3′UTR*	118366813–118366915	(TTTTA)_7_(TTA)(TTTTA)_13–exp_	(TTTTA/TTTCA)_440–3,680_	[23]
16	*TNRC6A*	1	24703039–24703078	(TTTTA)_8_	(TTTTA/TTTCA)_exp_	[23]

Chr.: chromosome; Ref.: reference; *: location varies depending on the isoform

## Epilepsy

Seizure onset age is variable, ranging between 12 and 67 years, with a mean age at onset of 30 years [13]. In FAME1 and FAME2, seizures usually start a few years after myoclonus onset, whereas in patients with expansions in the *MARCHF6* gene (FAME3), seizures may occur before or at the same time as cortical myoclonus in some family members [7]. In some FAME pedigrees, anticipation has been observed, at least for the age at epilepsy onset [7], [14], [15]. As in other primary generalized epilepsies (GE), seizures in FAME patients are commonly exacerbated or triggered by photic stimulation, alcohol or sleep deprivation [16]. Seizures tend to be less frequent compared to other forms of GE, but there are reports of intractable courses, which require a poly-antiepileptic drug regimen [5]. Absences are rare but were described in an Italian pedigree [17]. Epilepsies with a FAME etiology must be distinguished from juvenile myoclonic epilepsy (JME) and progressive myoclonus epilepsy when tremor is not a key distractor. In JME, the myoclonic-like seizures often occur in teens or young adults during wakeup in the morning. The deleterious progression and the occurrence of ataxia and dementia is a hallmark of progressive myoclonus epilepsy and will help to differentiate from FAME during the further time course. More rarely, other symptoms may be present in FAME patients including cognitive decline or intellectual disability, migraine and night blindness [4], [13].

## Electrophysiology and brain imaging

Cortical myoclonus is generated by abnormal neuronal discharges in cortical layers. The discharges lead to a brief activation of the corticospinal tract. In contrast, negative myoclonus is characterized by a sudden pause of corticospinal tract activity. A negative myoclonus is also known as asterixis or “flapping tremor.”

The electrophysiology of cortical myoclonus is based on electromyographical measurements (EMG), somatosensory-evoked potentials (SSEP) and long latency stretch reflexes (LLSRs). In the EMG, the distinction is made based on the frequency spectrum and the duration of the EMG burst. In patients with a central tremor component (e. g., ET, Parkinson’s disease) a synchronized specific frequency with a strong peak in the EMG power spectrum is observed. In contrast, the EMG of myoclonus reveals arrhythmic bursts of variable duration. The irregularity with short burst-like potentials will help to guide the clinician toward a myoclonus, which is often stimulus-sensitive. The burst duration in cortical myoclonus is shorter (<100 ms) compared to subcortical generated myoclonus.

Backaveraging of simultaneous EMG and EEG can detect that cortical discharges on EEG precede the jerks seen on EMG [18]. The SSEP may present giant cortical responses [19] as another sign of the cortical origin of the myoclonus. In addition, a reduction in the resting motor threshold intensity and the post-motor-evoked potential silent period has been reported in a few patients evaluated by transcranial magnetic stimulation, indicating that central motor inhibitory mechanisms are impaired in these cases [20]. Interestingly the cerebellum seems to play a role in the generation of cortical myoclonus via cerebello-thalamo-cortical projections that could change the gain of sensorimotor connections [21]. Long loop responses called C-reflexes which are elicited at rest are often detectable in FAME patients, indicating an increased cortical excitability [13].

Brain imaging studies, especially MRI, regularly did not show any consistent abnormalities. Proton magnetic resonance spectroscopy has reported an elevated choline/creatine ratio in the cerebellum cortex of patients from an Italian pedigree linked to chromosome 2 (FAME2) compared with controls [22]. Interestingly, *STARD7* encodes a member of the StAR-related lipid transfer (START) domain-containing family of lipid transfer proteins whose functions include intra-mitochondrial lipid transfer of phosphatidylcholine [14]. So far, it is not clear if increased choline/creatine ratios are restricted to FAME2 patients, but if so, this could indicate an alteration of the mitochondrial function of STARD7 in brains of FAME2 subjects.

## Current treatment

Since there are only anecdotal data on pharmacological interventions in FAME available, treatment options rely on general guidelines for symptomatic treatment of cortical myoclonus [9], which overlaps with treatment for epilepsies. First line treatment should include levetiracetam, sodium valproate and/or benzodiazepines.

## Genetic basis: a dominant disorder resulting from the same noncoding repeat expansion in at least six different genes

Four genetic loci for FAME have been identified by linkage analysis (2p11-2q11, 3q26-q28, 5p15, 8q24) in the past 20 years but the underlying genetic cause remained unknown despite extensive sequencing of genes in the candidate intervals. The decisive breakthrough was achieved when intronic repeat expansions in sterile alpha motif domain containing 12 (*SAMD12*) (FAME1) were identified as the main cause of BAFME/FAME in Japan [23]. This accomplishment was possible thanks to the reduction of the candidate interval on chromosome 8 to a single gene. Expansions composed of two different pentamer repeat motifs, TTTTA, which is the motif normally present at this locus, and TTTCA, which corresponds to an insertion, were identified based on non-Mendelian inheritance of the corresponding region due to the nonamplification of the expanded allele using standard PCR conditions [23]. *SAMD12* expansions are associated with a specific haplotype originating from a common Asian ancestor and account for a large proportion of FAME families in China, India and Sri Lanka [8], [23], [24], [25], [26]. In two Japanese families without *SAMD12* expansions, similar expansions in two other genes, *TNRC6A* on chromosome 16 (FAME6) and *RAPGEF2* on chromosome 4 (FAME7), were identified in a small family and a sporadic case, respectively [23]. A pathogenic expansion in *RAPGEF2* was further identified in another FAME family of Chinese origin [24].

**Figure 1:**
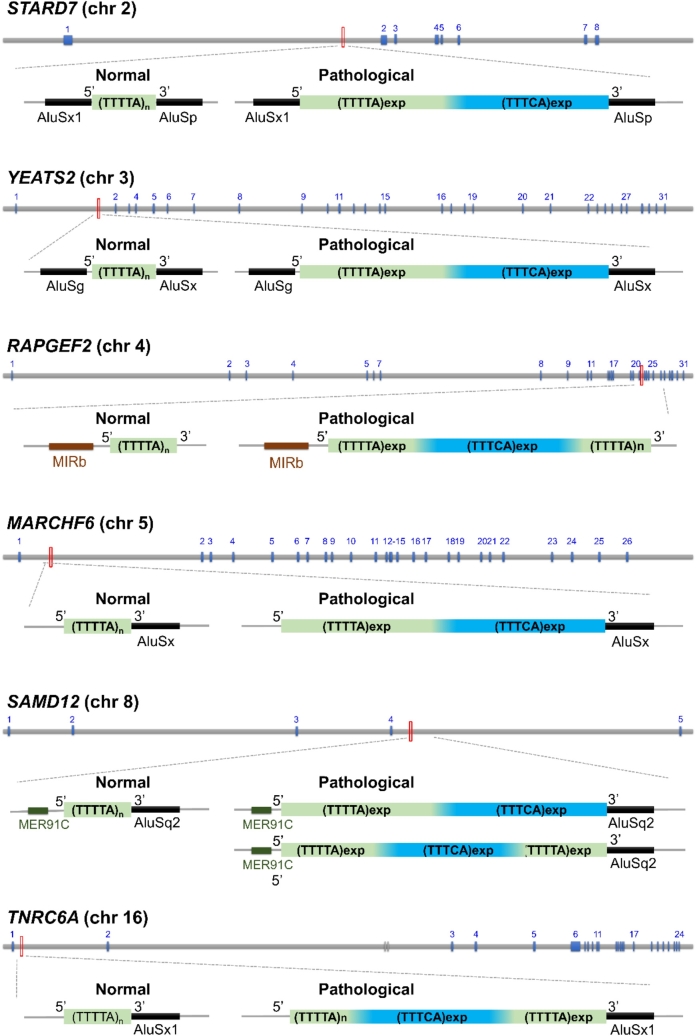
FAME is caused by similar pentameric repeat expansion in six different genes (*STARD7*, *YEATS2*, *RAPGEF2*, *MARCHF6*, *SAMD12*, *TNRC6A*) located on different chromosomes. These expansions always occur at a polymorphic short tandem repeat (microsatellite) site initially composed of TTTTA motifs (in green) in healthy individuals (on the left). This microsatellite is usually adjacent to one or more Alu elements. Pathogenic repeat expansions (on the right) always include a TTTCA repeat insertion (in blue) that likely corresponds to the disease-associated part of the expansions and is mainly located at the 3′ end of the expansion. Configurations with this insertion in a more central position have also been described in *SAMD12*. All expansions are intronic and are likely transcribed as part of pre-mRNA transcripts. They could lead to a toxic RNA able to accumulate and trap RNA-binding proteins, although this remains to be demonstrated.

The identification of *SAMD12* expansions in FAME1 was followed by similar discoveries at the three other loci: TTTTA/TTTCA expansions were identified in introns of *STARD7* on chromosome 2 [14], *MARCHF6* (formerly *MARCH6*) on chromosome 5 [7] and *YEATS2* on chromosome 3 [27]. Altogether, similar intronic TTTTA/TTTCA repeat expansions have thus far been identified in six different genes (Fig. 1, Table 2). In all genes, expansions replace a polymorphic primate-specific microsatellite originally composed of TTTTA repeats, usually adjacent to one or more Alu elements (Fig. 1). The size of the expansions is variable, ranging from 2 to 18 kb with an average of 4–5 kb (i. e., 800–1,000 repeats). There exists an inverse correlation between the age at onset and the overall size of the expansion [7], [23], which is mainly determined by the length of the TTTCA repeats [7]. Expanded *SAMD12* alleles exclusively composed of TTTTA repeats exist in a subset of healthy Japanese individuals, which further suggests that TTTCA repeats constitute the pathogenic part of the expansion while TTTTA repeats are nonpathogenic [23].

**Figure 2:**
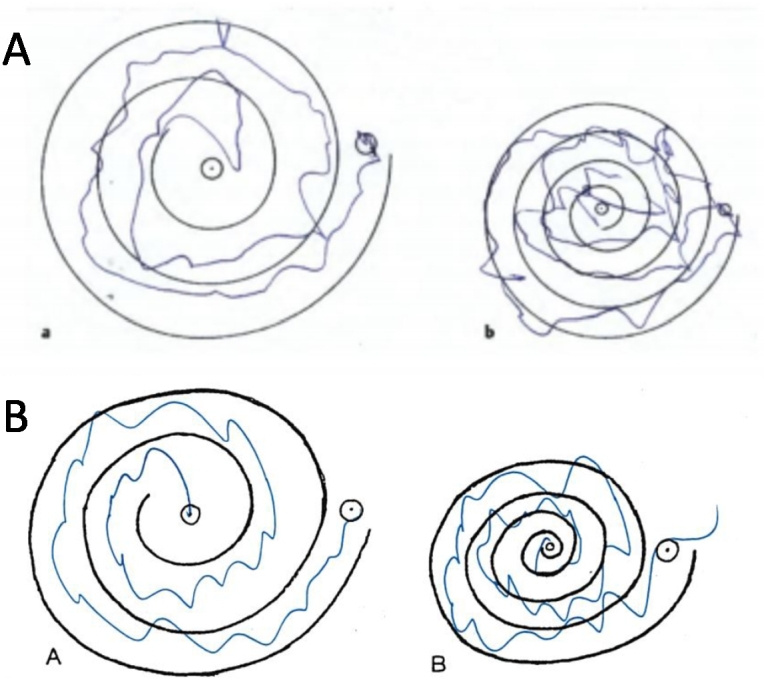
Archimedes spiral test of a FAME patient (A) (from Florian et al. [7]) and a moderately affected patient with ET (B). The figure shows the poor clinical differentiation between cortical myoclonus (FAME; A) and tremor (ET; B).

FAME repeat expansions are highly unstable in a length-dependent manner. The characterization of *MARCHF6* expansions using molecular combing (fiber FISH) has revealed that individual expanded alleles vary considerably in length and structure, with expansion size ranging from 1.7 to 36 kb in blood cells of a same individual. Furthermore, expansions exceeding 10 kb, present in individuals with the most severe phenotypes, were associated with microrearrangements at the site of the expansion, representing up to 10 % of alleles with an expansion [7]. These results suggest that FAME expansions constitute fragile sites prone to chromosomal breakage.

Interestingly, a large Chinese FAME family carrying a *SAMD12* expansion composed of a TTTGA insertion instead of the TTTCA repeats was reported, suggesting that other motifs than TTTCA could also be pathogenic [28]. However, a recent study has shown that TTTCA and TTTGA insertions coexist in some expansions [29], and therefore sequencing of the full expansion by a long-read technology taking the high degree of mosaicism into account is of utmost importance.

## Diagnostic work-up

The clinical diagnosis of FAME is based on: (1) the patient’s clinical history, which is usually suggestive of autosomal dominant inheritance; (2) the association of cortical myoclonus and seizures, either in the patient or within the family; and/or (3) a cortical origin of the tremor demonstrated by backaveraging of simultaneous EMG and EEG. However, this diagnosis should also be considered for patients without family history as sporadic cases have also been occasionally reported. ET and JME, which are the two main differential diagnoses, should be considered and ruled out based on seizure semiology or electrophysiological investigations (EEG, EMG and SEPs).

Identification of the molecular cause both allows to confirm the clinical diagnosis and enables genetic counseling in the family. Genetic testing usually requires a blood sample used to extract genomic DNA and perform repeat-primed PCR (RP-PCR) assays for the three main loci (FAME1, FAME2, FAME3). Alternative technologies, including molecular combing, nanopore sequencing and/or optical genome mapping, may also be used although these techniques are so far often limited to research studies. Repeat expansions linked to FAME can also be detected from genome data using specific bioinformatics tools such as ExpansionHunter [30]. However, a confirmation that the expansion includes pathogenic TTTCA repeats using either RP-PCR or a sequencing technique is recommended.

## Pathophysiological mechanisms leading to FAME

The identification of similar pentameric expansions in introns of unrelated genes strongly suggests that the pathological mechanisms are independent of the gene or its function. This hypothesis is further supported by the completely different functions and expression profiles of the six genes where FAME expansions occur: *SAMD12* is a gene of unknown function encoding three differently spliced isoforms, the largest one being predominantly expressed in the cortex and cerebellum [23]. Rap guanine nucleotide exchange factor 2 (*RAPGEF2*) encodes several isoforms primarily expressed in neurons, with highest expression in the cortex. These proteins belong to the RAS subfamily of GTPases and act to switch on Ras and/or ERK signaling pathways in response to the activation of cell surface receptors, such as dopaminergic receptors [31], [32]. Trinucleotide repeat-containing gene 6A (*TNRC6A*), also called glycine/tryptophane repeat protein (GW182), encodes a component of a cytoplasmic ribonucleoprotein complex involved in the regulation of mRNA silencing, stability and translation that is ubiquitously expressed, with highest expression in the cerebellum [33], [34]. *MARCHF6* encodes a ubiquitously expressed E3 ubiquitin ligase that mediates the degradation of misfolded or damaged proteins in the endoplasmic reticulum [35], [36]. *STARD7* encodes a ubiquitous protein involved in lipid transport and metabolism [37]. *YEATS2* encodes a ubiquitous subunit of the ADA2A-containing histone acetyltransferase complex, with high expression in the cerebellum [38]. Some genes are specifically expressed in the central nervous system, while others are ubiquitously expressed, but all six are expressed in the human brain, including the cortex and the cerebellum.

Since expansions are all intronic, expanded repeats should be transcribed as part of precursor mRNA (pre-mRNA) transcripts. However, contradictory results have been obtained regarding the expression of UUUUA/UUUCA repeats. On the one hand, reads corresponding to *SAMD12* transcripts filled with UUUUA/UUUCA repeats have been detected in association with RNA foci and abortive transcription in post-mortem brains of Japanese patients [23]. On the other hand, despite the ubiquitous expression of *MARCHF6* and *STARD7*, no reads filled with UUUUA or UUUCA repeats could be detected in lymphoblasts or fibroblasts of patients with expansions in these genes [7], [14]. Furthermore, the expression and splicing of *MARCHF6* and *STARD7* were unaltered in these tissues compared to unaffected controls, and there was no increase in *MARCHF6* intron 1 retention, which would be expected if abortive transcription and RNA accumulation would occur [7], [14]. These discrepant results suggest that abnormal transcription and accumulation of UUUCA repeats observed in *SAMD12* expansion carriers could be restricted to neuronal tissues or do not exist in other FAME subtypes, and additional studies are required to determine whether RNA foci exist in other FAME subtypes.

Remarkably, TTTTA/TTTCA expansions in intron 3 of *DAB1* have initially been associated with spinocerebellar ataxia 37 (SCA37) [39]. *DAB1* encodes a downstream effector of the reelin signaling pathway, contributing to the correct positioning of neurons in the developing brain. Its expression is higher in the cerebellum than in the cortex, possibly explaining why expansions at this locus are specifically associated with cerebellar ataxia. However, several FAME-associated genes (e. g., *TNRC6A*, *YEATS2*) are also highly expressed in the cerebellum, suggesting that the expression of the genes where expansions occur is not the only factor influencing the phenotypic presentation. A recent study based on post-mortem brains of SCA37 expansion carriers revealed that SCA37 expansions increase *DAB1* expression and trigger alternative splicing events favoring the inclusion of two exons absent from isoforms normally present in the brain [40]. Yet, SCA37 and FAME expansions appear to have different characteristics: FAME TTTCA repeats are predominantly located at the 5′ extremity of the expansion, whereas SCA37 TTTCA repeats are inserted in the middle of TTTTA repeats. SCA37 expansions also tend to be shorter in size (usually up to 1 kb), while the shortest FAME expansion described so far was above 2 kb. Interestingly, nonpathogenic expansions up to 3 kb composed of TTTTA repeats only also exist at the *DAB1* locus, further confirming that homogeneous TTTTA expansions are benign, independently of the locus.

## Conclusions

Recent genetic evidence revealed that FAME is a repeat expansion disorder involving the same AT-rich pentameric motifs in distinct genes. The description of pedigrees without expansions in one of the six known loci argues for the existence of additional loci of this repeat expansion [7]. Further research studies are necessary to apprehend the mechanisms by which these expansions are pathogenic but the observation of RNA foci in FAME1 [23] argues for possible RNA-mediated toxicity that remains to be proven in other FAME subtypes. Due to its rarity and overlap with more frequently occurring neurological disorders, FAME patients are likely often misdiagnosed. Diagnostic consensus criteria for FAME remain to be redefined as, to our knowledge, the available criteria have been proposed in 2005 and 2006 [41], [42], i. e., long before the identification of its genetic cause.
